# MR‐linac MLC positioning QA by digitally stitching dual double‐exposed films

**DOI:** 10.1002/acm2.14325

**Published:** 2024-03-11

**Authors:** Sheng‐Hsuan Sun, Erika Kollitz, Wen‐Chih Tseng, Amanda Schwarz, Luke Maloney, Jonathan Li, Chihray Liu, Guanghua Yan

**Affiliations:** ^1^ Department of Radiation Oncology University of Florida Gainesville Florida USA

**Keywords:** Elekta Unity, MLC positioning accuracy, MR‐linac, Picket Fence test

## Abstract

**Purpose:**

The picket fence (PF) test is highly recommended for multi‐leaf collimator (MLC) quality assurance. However, since the electronic portal imaging device (EPID) on the Elekta Unity only covers a small area, it is not feasible to perform the PF test for the entire MLC. Here, we propose a technique for the PF test by stitching two double‐exposed films.

**Methods:**

Two EBT3 films were used to encompass the entire MLC, with each one covering one half of the area. Two fields were employed to apply double exposure: a PF pattern consisting of 11 2 mm wide pickets and a 2.84 cm x 22 cm open field. The edges of the open field defined by the diaphragms were used to correct film rotation as well as align them horizontally. The PF pattern was also measured with the EPID where the pickets were used to align the films vertically. Individual leaf positions were detected on the merged film for quantitative analysis. Various MLC positioning errors were introduced to evaluate the technique's sensitivity.

**Results:**

The merged films covered 72 leaf pairs properly (four leaf pairs on both sides were outside the treatment couch). With the EPID, the leaf positioning accuracy was ‐0.02 ± 0.07 mm (maximum: 0.29 mm) and the picket width variation was 0.00 ± 0.03 mm (maximum: 0.11 mm); with the films, the position accuracy and width variation were ‐0.03 ± 0.13 mm (maximum: 0.80 mm) and 0.00 ± 0.13 mm (maximum: 0.74 mm), respectively. The EPID was able to detect errors of 0.5 mm or above with submillimeter accuracy; the films were only able to detect errors > 1.0 mm.

**Conclusion:**

We developed a quantitative technique for the PF test on the Elekta Unity. The merged films covered nearly the entire MLC leaf banks. The technique exhibited clinically acceptable accuracy and sensitivity to MLC positioning errors.

## 1 INTRODUCTION

Magnetic resonance (MR)‐guided radiotherapy (MRgRT) is an emerging technology that combines real‐time MR imaging with high‐precision radiation delivery.^1^ It offers two key advantages over conventional image‐guided radiotherapy (IGRT) systems, that is, superior soft‐tissue contrast without radiation exposure to the patients, and the ability of online adaptive treatment planning and delivery.^2^ On the other hand, MRgRT also poses new challenges for system quality assurance (QA), due to the complex interactions between the MR and the linear accelerator (linac) components, and the design change from conventional linac.^3^ This study addresses a specific issue related to the QA of the multi‐leaf collimator (MLC) positioning accuracy in an MR‐linac system.

The key component for intensity modulation in modern linacs is the MLC, which shapes the radiation beam according to the target volume and protects the surrounding normal tissues. The accuracy and stability of the MLC positioning are essential for ensuring the quality and safety of the treatments. The picket fence (PF) test and the strip pattern test are the typical tests to evaluate the MLC positioning accuracy.^4^, ^5^, ^6^ In such tests, various special MLC patterns are designed to provide an assessment of the positions of individual MLC leaves and in relation to the alignments of the other leaves. On conventional linacs, these tests are routinely performed with either film or the electronic portal imaging device (EPID).

However, on the commercial MR‐linac system, Elekta Unity (Elekta AB, Stockholm, Sweden), these tests become challenging. The maximum field size of Unity is 57.4 cm x 22.0 cm, which is larger than the size of a typical EBT3 film. For example, the sheet dimension of a GAFCHROMIC EBT3 film that is widely used in radiotherapy QA is only 43.2 × 32.5 cm. Therefore, a single EBT3 film is not large enough to cover the entire field available on Unity. Meanwhile, the EPID on the system (also referred to as the megavoltage imager, or MVI) has a field view of only 22.0 cm x 9.5 cm, limited by the cryostat gap separating the main MR coils that generate the static magnetic field.^7^ As a result, the EPID only covers a limited portion of the leaf bank in the center. To overcome these limitations, Snyder et al. designed a film holder to capture the entire leaf bank with the diagonal of a single film.^8^ To that end, the film has to be brought closer to the MLC, which is limited due to the closed bore of the MR system. Consequently, the film holder has an inclined surface. In their design, the film can cover the entire leaf bank, but the covered range along the leaf moving direction is limited to the central 2 cm.

The aim of this paper is to present a quantitative method for MR‐linac MLC positioning QA, utilizing commonly available materials found within clinical settings. The proposed approach employs two EBT3 films to encompass the complete MLC leaf banks and their entire moving range. Each film spans half of the leaf bank (in the direction perpendicular to leaf movement) and undergoes double exposure with two fields: a PF MLC pattern and an open field. The two films are scanned and digitally stitched together, with the open field serving as the reference for registration. The two fields are also measured with the EPID and analyzed to help film registration. Subsequently, individual leaf positions are extracted from the merged film for quantitative analysis of their positioning accuracy. This paper describes and characterizes the technique, with a comparison against the PF test with the EPID for the central leaf pairs. We also examined the technique's sensitivity to intentionally introduced MLC positioning errors.

## 2 METHODS AND MATERIALS

### 2.1 MR‐linac MLC system

Zhang et al. provided a detailed description of the MLC system on Elekta Unity and thoroughly evaluated its performance.^7^ The system is based on the Agility model and customized for MR‐linacs. It has 80 leaf pairs that move only along the IEC 61217 Y‐axis (patient superior to inferior direction) since diaphragm rotation is prohibited on Unity. The diaphragm movement is parallel to the IEC 61217 X‐axis (patient left to right direction). The nominal leaf width at a source axis distance (SAD) of 143.5 cm is 0.7175 cm, which defines the largest field width of 57.4 cm. Because of the cryostat gap, the leaf travel range is only 22 cm along the Y‐axis. The EPID, located at 265.3 cm from the source, has a pixel size of 0.04 cm and a field of view of 21.0 cm x 8.5 cm as projected at SAD. As a result, only the central 30 leaf pairs can be covered by the EPID within an 8.5 cm range along the Y‐axis.

### 2.2 The proposed technique

**FIGURE 1:**
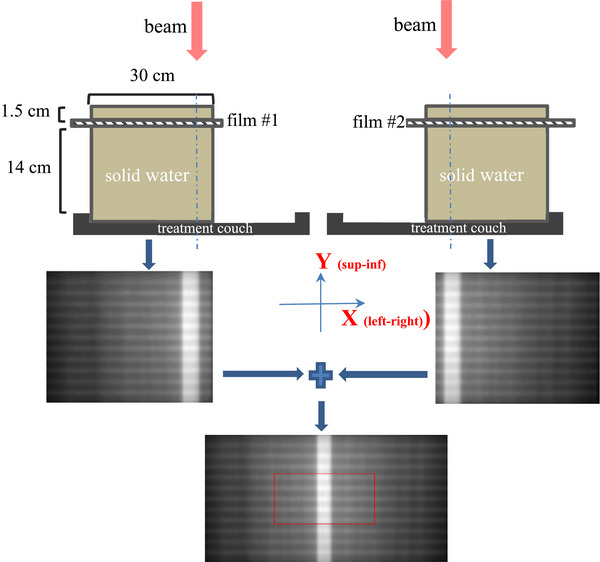
The proposed technique uses two EBT films to cover the entire MLC leaf bank, with each film covering a half of the area. Each film was irradiated with two fields: (1) a picket fence pattern consisting of 11 2 mm wide pickets (2) a 2.84 cm x 22 cm open field. The two films were digitally stitched together using the open field for registration. Individual leaf positions were extracted from the merged film for quantitative analysis. In the image at the bottom, the region indicated by the red rectangle represents the 21.0 cm x 8.5 cm area that can be covered by the EPID on the Elekta Unity.

The proposed technique attempts to cover all the leaf pairs and their entire travel range with two EBT3 films (Figure 1). Film measurements were performed in 30 cm x 30 cm solid water with a 1.5 cm build‐up and 14 cm backscatter, which placed the film at the isocenter plane. For the first film (film #1), we aligned the solid water phantom against the edge of the treatment couch on the ‐X side (patient right), and placed the film with its center aligned with the center of the solid water. A PF pattern was delivered, followed by a vertical open field. For the second film (film #2), we aligned the solid water phantom against the edge of the couch on the +X side (patient left). We reversed the order of the beam delivery, that is, the open field was delivered first, followed by the PF pattern. In this way, we guaranteed that there was no diaphragms or MLC leaf motion between the two deliveries of the open field since it would be used to register the two films.

The vertical open field had a size of 2.84 cm x 22 cm with 300 MUs. The PF MLC pattern consisted of 11 pickets of 2 mm width. A 57.4 cm x 2.0 cm horizontal field was delivered in the step‐and‐shoot mode, starting from near the field edge on the +Y axis. At each step, the entire open field was shifted towards the ‐Y direction by 1.8 cm, creating a 2 mm over‐exposed strip. The rectangular field was delivered 12 times with 300 MUs each to create 11 pickets, with the middle one centered on the X axis. As a result, the separation between adjacent pickets was 1.8 cm.

Additionally, the PF MLC pattern and the open field were measured separately with the EPID. These measurements were used to aid film registration and validate the film results, albeit only the central five pickets covering the central 30 leaf pairs were available from the EPID measurement (Figure 1). In summary, the PF pattern and the open field (OF) were delivered in this order: (1) PF to the EPID (2) PF to film #1 (3) OF to film #1 (4) OF to the EPID (5) OF to film #2 (6) PF to film #2 (7) PF to the EPID. All the deliveries were performed at 0° gantry angle. The PF pattern was measured with the EPID at the beginning and the end to assess short‐term MLC positioning reproducibility. Both films received dual‐exposure (the PF pattern and the open field). The open field was delivered consecutively in steps 3, 4, and 5 to ensure there was no jaw or MLC leaf motion in between.

### 2.3 Film stitching and data analysis

The films were scanned at least 2 h after irradiation with an EPSON Expression 10000XL flatbed scanner (Seiko Epson Corporation, Nagano, Japan). The manufacturer of EBT3 film recommends to wait 1−2 h after exposure before scanning to reduce the impact of post‐exposure density growth, especially in absolute dosimetry applications. While our application did not involve absolute dosimetry, we took a conservative approach to ensure the highest level of accuracy in our results. The films were placed in the center of the scanner bed in the landscape orientation (the longer side of the films was parallel to the scanner bed). The scanned images were processed with in‐house Matlab scripts. Three steps were taken to stitch the two films together:

(1) Rotation correction to both films. The vertical edge (X jaw) of the open field was located on each film and the rotation was calculated by fitting a line to the points on the edge. Since collimator rotation is forbidden on the Unity, the X jaw remains parallel to the MLC motion direction and could be used to correct film rotation.
(2) Horizontal alignment of the films. The open field was also used to align the two films in the horizontal direction. Since the open field was symmetric, the isocenter in the horizontal direction (x‐coordinate) was placed in the middle of the two vertical edges on both films.
(3) Vertical alignment of the films. On each film, a vertical profile along the center of the open field was extracted after the rotation was corrected. The peaks of the five pickets in the middle (#4‐#8) were located. These peaks were used to calculate a mean shift needed to align the two films vertically. Only the middle five pickets were used for this purpose since they were also available on the EPID measurement, which could be used for validation. In this process, the y‐coordinate of the isocenter was also transferred from the EPID to the film.

**FIGURE 2:**
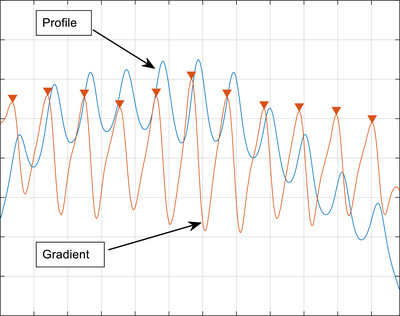
Individual leaf positions were calculated with the gradient of the vertical intensity profile crossing the center of each leaf pair. For each picket, the location with local maximum gradient (marked with an inverse triangle) was regarded as the leaf position of one leaf; the location with local minimum gradient (valleys of the gradient curve, not marked) was regarded as the position of its opposing leaf.

On the merged film, individual leaf positions were detected for analysis. Specifically, vertical profile along the center of each leaf pair was extracted and the gradient of each point was calculated. Leaf positions were identified as the points with either local maximum or minimum gradient (Figure 2). The gradient method was chosen over the method using full‐width at half maximum since it disregards the intensity variation due to the lack of the flattening filter on Unity.^6^, ^9^ For each leaf pair, we evaluated the deviation of the picket position (defined as the center of each picket) from the nominal value, and the variation of the picket width. Similar analysis was also performed for the two EPID measurements of the PF pattern, the results of which were used to assess short‐term reproducibility of the MLC leaves and validate the film results.

### 2.4 Sensitivity to MLC positioning errors

To test the technique's sensitivity to MLC positioning errors, various MLC positioning errors were introduced into the PF pattern. For the first and last picket, a 1.0 mm error was introduced to the leaf pair #40, and a 2.0 mm error was introduced to leaf pairs #25 and #55. A 0.5 , 0.7 , 1.0 , 1.5 , and 2 mm error was introduced to leaf pair #40 for the five pickets in the center (from +Y to ‐Y), respectively. This PF pattern was delivered and analyzed with the process described above.

## 3 RESULTS

**FIGURE 3:**
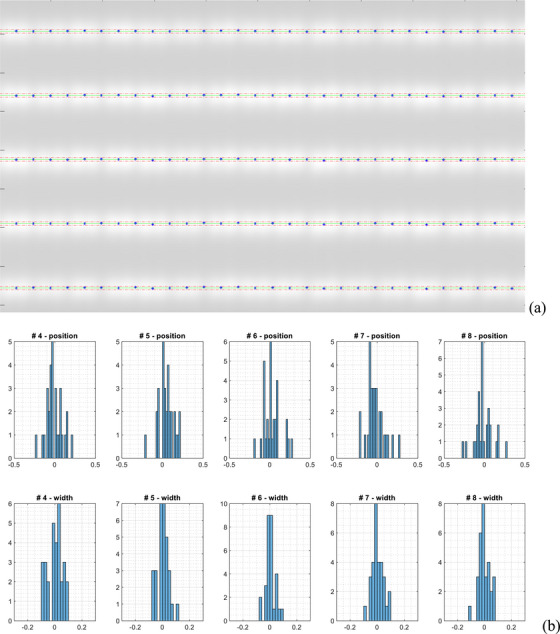
Results of the picket fence test with the EPID on Unity (a) detected MLC positions (blue stars) in relation to nominal values (green line) and a ± 0.5 mm tolerance (red lines). (b) Top—histogram of the MLC position deviation from their nominal values (unit: mm); bottom—histogram of the picket width variation (unit: mm).

Figure 3 shows the results from the first EPID measurement of the PF pattern. In Figure 3a, the detected picket positions are presented alongside their nominal values and a ± 0.5 mm tolerance. Figure 3b shows the histogram of the deviations of the picket positions and widths for each picket (30 leaf pairs). The average deviation of the picket positions from their nominal values was 0.02 ± 0.07 mm with a maximum of 0.29 mm; the average variation of the picket widths was 0.00 ± 0.03 mm with a maximum of 0.11 mm. In the second EPID measurement, the average picket position deviation was 0.01 ± 0.07 mm with a maximum of 0.27 mm and the average picket width variation was 0.00 ± 0.03 mm with a maximum of 0.12 mm. These results show that the MLC leaves that were covered by the EPID had excellent positioning accuracy and short‐term reproducibility.

**FIGURE 4:**
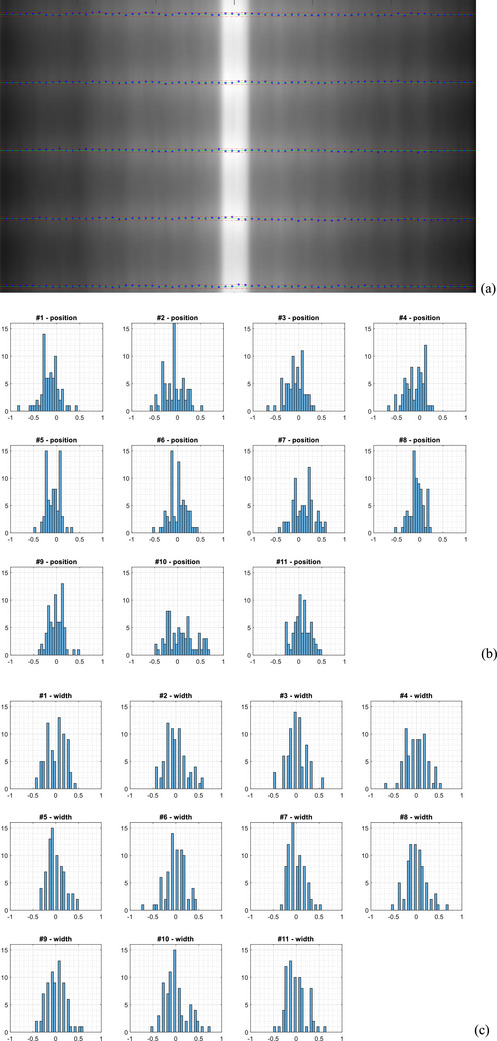
Results of the picket fence test with the films (a) detected MLC positions (blue stars) in relation to nominal values (green line) and a ± 0.5 mm tolerance (only the five pickets in the film center were presented here for better readability). (b) Histogram of the MLC position deviation from their nominal values (unit: mm). (c) Histogram of the picket width variation (unit: mm).

Figure 4 shows the results from the film measurement. To improve readability, only the five pickets in the center (#4 to #8) of the merged film are presented in Figure 4a. Each picket included only 72 leaf pairs. The four outmost leaf pairs on both sides covered area outside the solid water phantom which led to improper film exposure, so their results are excluded from the analysis below. The mean picket position deviation was −0.03 ± 0.13 mm (maximum: 0.8 mm) and 2% of the positions had deviations greater than 0.5 mm. The mean picket width variation was 0.0 ± 0.13 mm (maximum: 0.74 mm) and 2% of the widths had deviation greater than 0.5 mm.

**FIGURE 5:**
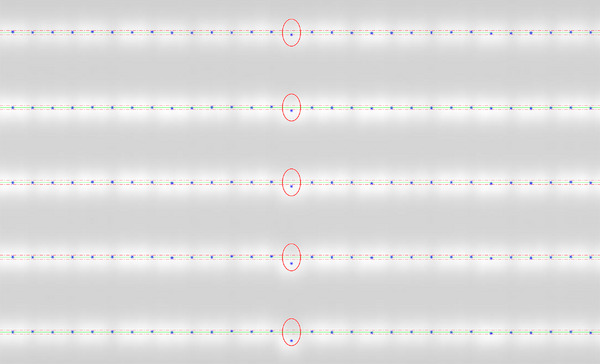
Sensitivity of the picket fence test with the EPID to MLC positioning errors of 0.5 , 0.7 , 1.0 , 1.5 , and 2.0 mm (from top to bottom, indicated with red circles). The detected deviations from their nominal values were 0.52 , 0.73 , 0.98 , 1.48 , and 2.06 mm.

The sensitivity of the PF test to MLC positioning errors with the EPID is illustrated in Figure 5. When the introduced errors were 0.5 , 0.7 , 1.0 , 1.5 , and 2.0 mm (from top to bottom, indicated with red circles), the detected deviations were 0.52 , 0.73 , 0.98 , 1.48 , and 2.06 mm, respectively.

**FIGURE 6:**
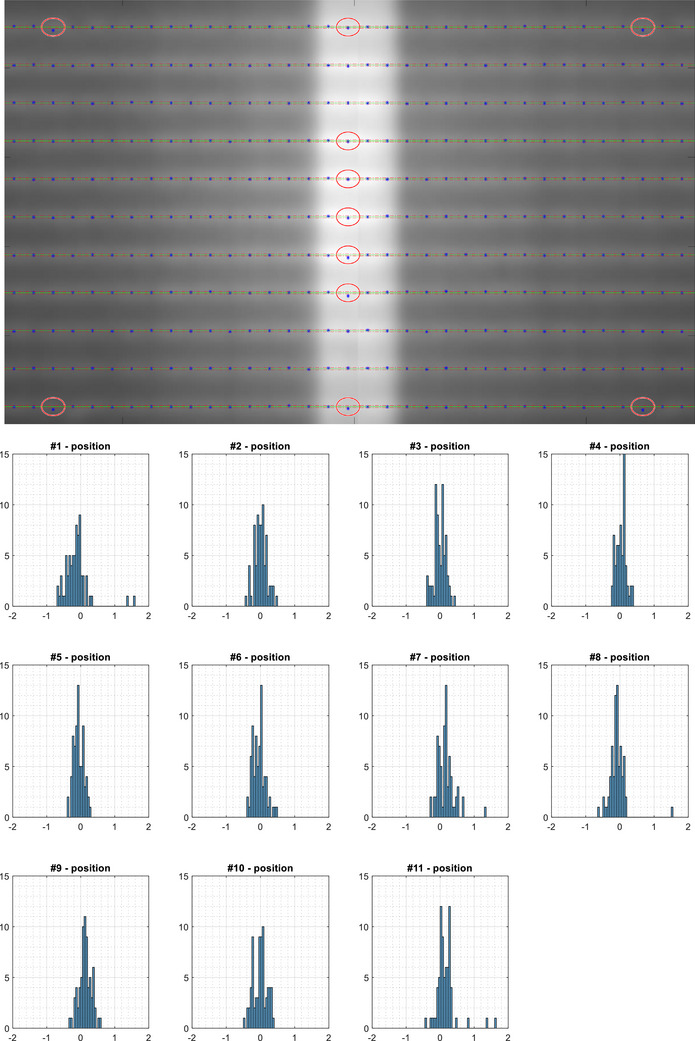
Sensitivity of the picket fence test with the films to MLC positioning errors. Top—detected picket positions vs. nominal values. The red circles indicate where positioning errors were introduced; Bottom—histogram of the detected picket positions for each picket (unit: mm).

The sensitivity of the PF test to MLC positioning errors with film is illustrated in Figure 6. In Figure 6a, only leaf pairs #23 through #57 were displayed for better visibility. For the leaf pairs with 2.0 mm error, the detected picket position deviations were 1.45 mm (leaf pair #25 of picket #1), 1.60 mm (leaf pair #55 of picket #1), 1.55 mm (leaf pair #40 of picket #8), 1.35 mm (leaf pair #25 of picket #11), and 1.60 mm (leaf pair #55 of picket #11). For the only leaf pair with 1.5 mm error, the detected picket position deviation was 1.3 mm (leaf pair #40 of picket #7). For the other six positioning errors no more than 1.0 mm, the detected deviation was under 0.7 mm except for leaf pair #40 of picket #11, where the detected deviation was 0.82 mm.

## 4 DISCUSSION

Periodical PF test is highly recommended for MLC QA in advanced radiotherapy, especially for MR‐guided precision radiotherapy, where high dose often needs to be delivered to relatively small off‐center targets. On the Elekta Unity, the complete PF test could not be performed with the EPID due to its limited size. In this study, we developed a simple technique to address the issue. The technique uses materials that are readily available in most clinics (i.e., EBT3 films and solid water phantom).

The main idea of the technique is to use two films to cover the entire field and digitally stitch them together for quantitative analysis. The challenges include film rotation correction and alignment in both horizontal and vertical directions. Unlike conventional linac, the Elekta Unity does not have laser or light field to guide film placement. We meticulously aligned both films to ensure their edges were parallel to those of the solid water phantom, which could still result in an unavoidable albeit small rotation. Additional rotation could have been introduced during film scanning. Here we used an open 2.84 cm x 22 cm field for a double expose on the films to address the problem. To minimize the impact on leaf position detection, we chose an open field that spanned exactly four leaves with both diaphragms fitting between two adjacent leaves. The longest possible field was chosen to achieve best possible accuracy for rotation correction. This open field was also used to locate the x‐coordinate of the isocenter on both films, which was used to align the two films in the horizontal direction. The open field was measured with the EPID to ensure the positioning accuracy of the diaphragms. The alignment of the two films in the vertical direction was achieved by aligning the five central pickets between the films and the EPID. The y‐coordinate of the isocenter was also transferred from the EPID to the films. The maximum picket positioning deviation detected by the EPID measurement before and after film measurement was both under 0.3 mm, which demonstrates the superior performance of the EPID and excellent MLC positioning reproducibility. Zhang et al also observed excellent short‐term and long‐term MLC positioning reproducibility with the EPID.^7^

Snyder et al also used the film to evaluate the leaf positioning accuracy on Elekta Unity, although their film could only cover 2 cm leaf motion range in the center.^8^ They observed similar mean deviations as ours (0.1 ± 0.19 mm vs. −0.03 ± 0.17), but their maximum deviation was smaller (0.5  vs. 0.8 mm). With the EPID, we also observed better results than with the films. The merged films covered nearly the entire treatment field, while the EPID only covered a smaller area in the center. This difference might have contributed to the difference in the results, but the main contributor could be the uncertainty associated with the film. The inherent variations in film response, film scanner characteristics, and environmental conditions could have affected the results.^10^, ^11^ These variations may also impact the sensitivity of the technique to artificially introduced positioning errors. While we could detect positioning errors of as small as 0.5 mm within sub‐millimeter accuracy with the EPID, we could only detect errors greater than 1.0 mm with the films. Due to the substantial level of noise, the film data was smoothed with a Gaussian‐weighted moving average filter, which smeared the introduced errors. Although the EPID and the film had different pixel sizes (0.216  vs. 0.085 mm), both were deemed adequate for our intended purpose.^7^

A limitation of the technique is that four leaf pairs on both sides could not be properly imaged by the films because they were outside the treatment couch. The signal in these areas of the film was further compromised by the electron return effect, rendering it useless for MLC leaf position detection. Although it is reasonable to assume that these MLC leaves will not be used to shape any treatment fields, qualitative check of their positioning accuracy could be performed by extending the film and the solid water outside of the treatment couch such that these leaf pairs could be covered by the film properly.

Due to the poor signal‐to‐noise ratio of the film, a large amount of MUs had to be delivered at each step. Since the Elekta Unity has a relatively low dose rate of 425 MU/min, it takes approximately 30 min to complete the entire measurements.

## 5 CONCLUSION

We developed a simple technique to address the challenge imposed by the limited size of the EPID during the PF test on the Elekta Unity. Dual double‐exposed films were digitally stitched together for quantitative analysis. It is the first quantitative PF test that covered nearly the entire MLC leaf banks across their full travel range on the Elekta Unity. We demonstrated that the MLC positioning accuracy of our Unity was within clinically acceptable range (< 1.0 mm). We also showed that the technique was able to detect MLC positioning errors of > 1.0 mm. The technique can be easily implemented since it uses only the EBT3 films and solid water phantom that are readily available.

## AUTHOR CONTRIBUTIONS

The authors collectively contributed to formulating the research concept and participated in the analysis of the results. Additionally, each author played a role in either drafting the manuscript or engaging in critical revisions.

## CONFLICT OF INTEREST STATEMENT

There is no conflict of interest.
